# Investigation of Host Candidate Malaria-Associated Risk/Protective SNPs in a Brazilian Amazonian Population

**DOI:** 10.1371/journal.pone.0036692

**Published:** 2012-05-16

**Authors:** Simone da Silva Santos, Taane G. Clark, Susana Campino, Martha Cecília Suarez-Mutis, Kirk A. Rockett, Dominic P. Kwiatkowski, Octavio Fernandes

**Affiliations:** 1 Laboratório Interdisciplinar de Pesquisas Médicas, IOC, Fiocruz, Rio de Janeiro, Brazil; 2 Faculties of Epidemiology and Population Health and Infectious and Tropical Diseases, London School of Hygiene and Tropical Medicine, London, United Kingdom; 3 Wellcome Trust Sanger Institute, Hinxton, Cambridge, United Kingdom; 4 Laboratório de Doenças Parasitárias, IOC, Fiocruz, Rio de Janeiro, Brazil; 5 Wellcome Trust Centre for Human Genetics, University of Oxford, Roosevelt Drive, Oxford, United Kingdom; Universidade Federal de Minas Gerais, Brazil

## Abstract

The Brazilian Amazon is a hypo-endemic malaria region with nearly 300,000 cases each year. A variety of genetic polymorphisms, particularly in erythrocyte receptors and immune response related genes, have been described to be associated with susceptibility and resistance to malaria. In order to identify polymorphisms that might be associated with malaria clinical outcomes in a Brazilian Amazonian population, sixty-four human single nucleotide polymorphisms in 37 genes were analyzed using a Sequenom massARRAY iPLEX platform. A total of 648 individuals from two malaria endemic areas were studied, including 535 malaria cases (113 individuals with clinical mild malaria, 122 individuals with asymptomatic infection and 300 individuals with history of previous mild malaria) and 113 health controls with no history of malaria. The data revealed significant associations (p<0.003) between one SNP in the IL10 gene (rs1800896) and one SNP in the TLR4 gene (rs4986790) with reduced risk for clinical malaria, one SNP in the IRF1 gene (rs2706384) with increased risk for clinical malaria, one SNP in the LTA gene (rs909253) with protection from clinical malaria and one SNP in the TNF gene (RS1800750) associated with susceptibility to clinical malaria. Also, a new association was found between a SNP in the CTL4 gene (rs2242665), located at the major histocompatibility complex III region, and reduced risk for clinical malaria. This study represents the first association study from an Amazonian population involving a large number of host genetic polymorphisms with susceptibility or resistance to *Plasmodium* infection and malaria outcomes. Further studies should include a larger number of individuals, refined parameters and a fine-scale map obtained through DNA sequencing to increase the knowledge of the Amazonian population genetic diversity.

## Introduction

Malaria is a life-threatening parasitic disease transmitted by mosquitoes. Despite major efforts aimed at controlling the spread and impact of the disease, it still persists as a major health burden, being responsible for over a million deaths each year, mainly children in Sub-Saharan Africa. In Brazil, there were over 300,000 recorded cases of malaria in 2010, almost exclusively (99.8% of the cases) restricted to the Amazon Basin region [1].

Malaria is a complex disease with many genetic and environmental determinants influencing the observed variation in response to infection, progression and severity. Several factors important for these different phenotypes include the parasite genetic make-up and host age, state of immunity and genetic background [2]. Resistance involves genetically-based and cell-mediated immunological mechanisms, including the production of specific antibodies that are main actors in the acquired immune response [3], thereby reducing the severity of symptoms and mortality. Resistance mechanisms have been described for both the liver and blood stages of the parasite in the host [4].

Significant associations have been described between malaria and a variety of host genetic polymorphisms that occur in erythrocytes and cells of the immune system. The different geographic distributions of sickle-cell disease, α-thalassemia, glucose-6-phosphate dehydrogenase (G6PD), southeast asian ovalocytosis and the Duffy-negative blood group are examples of the general principle that different populations have selected different genetic variants to protect against *Plasmodium* infection (see [5], [6], [7] for reviews). The sickle-cell trait (HbS) [8], G6PD (reviewed in [9]), and ABO blood group [10], are amongst a number of host genes with polymorphisms found to reduce the risk of severe malaria. Some genes relevant to immunity and inflammation, such as the tumor necrosis factor (TNF) within the MHC class III region, (reviewed in [11]), Toll-like receptors (TLR-4, TLR-9) [12], [13], CD40 ligand (CD40L) [14], interferon gamma (IFN-γ) (reviewed in [15]), and the nitric oxide synthase type 2 (*NOS2*A) genes (reviewed in [16]) have also been associated with severe malaria.

Previous genetic studies in Brazilian Amazonian populations have demonstrated different malaria protective effects from blood-related polymorphisms (e.g. Duffy, ABO, Rh, MNSs and Kell systems [17], [18], [19], [20]), erythrocyte enzymes (G6PD [18]), receptors (CR-1, complement receptor 1) [20], [21], and polymorphisms which play a critical role in the early innate immune response to invading pathogens (e.g. TLRs [20], [22]). Association of Duffy blood group gene polymorphisms and susceptibility to *P. vivax* malaria has been observed in five endemic states of the Brazilian Amazon [17], [18], [19]. Variants in the TLRs associated with clinical outcomes of malaria have been reported [20], [22]. In particular, a study across three areas of the Amazon basin found significant associations between TLR-1 and TLR-6 variants with mild malaria, whereas TLR-9 variants were associated with high parasitemia [22]. No association was found between TLR-4 polymorphisms and mild malaria [22]. These results are in agreement with previous studies that found no association between TRL-4 and protection to mild malaria in a community in the Baixo Amazonas region in the state of Pará [20]. Recent studies have also shown a possible association between CR1-polymorphisms and susceptibility to *P. falciparum* infection in individuals from an endemic area in the state of Amazonas [21]. However, in the state of Pará, no correlation was observed between CR-1 with resistance to *P. falciparum* infections [20]. It has been reported that the regulation of IL-10 levels, an anti- inflammatory cytokine, in *P. vivax* infected patients may be unaltered by polymorphism in the promoter region of IL-10 gene [23].

Here, we investigated the association of a larger number of host candidate genes polymorphisms with susceptibility/resistance to *Plasmodium* infection with clinical (mild) malaria in a population of the Brazilian Amazon. We genotyped 64 single nucleotide polymorphisms (SNPs) in 37 human host genes, including loci related with erythrocytes receptors and immune response. Our study is the first to comprehensively survey important malaria candidate polymorphisms (including HbS and ABO) in a Brazilian population from the Medium Negro River Basin in the Amazon. This setting provided an excellent field to test hypotheses on the generalization of established malaria-genetic associations, particularly for low endemic areas. It also provided a mean to find other associations related to either different mechanisms or by linkage disequilibrium with putative markers associated with susceptibility/resistance to *Plasmodium* infection [18].

## Materials and Methods

### Ethics Statement

Approval for the recruitment of participants, collection of blood samples, DNA preparation and DNA genotyping was provided by the relevant research ethics committee (Oswaldo Cruz Foundation - protocol number 360/06) and an informed consent was obtained from each participant.

### Study Participants

Potential participants were engaged between January 2002 and October 2006 from an ongoing epidemiological study of malaria in the cities of Barcelos (n = 596) and Santa Isabel do Rio Negro (n = 52), which are 350 km apart. The municipalities are located within the Negro river micro region in the state of Amazonas and display similar demographics. In 2006, the Annual Parasite Index (API) was 264.4 cases per 1000 inhabitants in Barcelos [24] and 127.2 cases per in Santa Isabel do Rio Negro [25]. The mean number of previous malaria episodes was 5.54±10.52 in Barcelos and 2.32±1.33 in Santa Isabel. These differences were not statistically significant (p = 0.053). Greater details of social, demographic and malaria data for the populations studied can be found elsewhere [25], [26].

The individuals included in this study had lived in the study area for at least five year with similar social and genetic backgrounds. The population of both municipalities is predominantly of Amerindian descent from Tukano-Oriental speaking societies [25], [27], [28]. Individuals were recruited during consultations for malaria symptoms at the health service centers in both cities. For each malaria case identified, a field team was dispatched to perform an active search of the patient’s house and neighboring houses. Healthy controls, asymptomatic infected individuals and individuals with a previous malaria history, but not infected at the moment of the study, were recruited during these active searches. A previous history of malaria in uninfected individuals and healthy controls was verified using reviews of health service charts from each municipality. Persons who had used anti-malarial drugs 30 days before the recruitment day were excluded. The participants had a median age of ∼19 years (malaria cases: median 18.0, range 1.0–88.0; controls 19.0, 3.0–72.0), and 48.5% were males (malaria cases: 283, 53.7%, controls: 31, 28.4%) (see Table 1).

**Table 1:** Baseline and clinical characteristics of the studied population.

	Controls (n = 113)		malaria cases (n = 535)	
	n (median)	% (range)	n (median)	% (range)
Age (years)	(19.0)	(3.0–72.0)	(18.0)	(1.0–88.0)
Gender (male)	31	28.4	283	53.7
Number of individuals				
Barcelos	102	90.3	494	92.3
Santa Izabel do Rio Negro	11	9.7	41	7.7
Clinical phenotype of malaria cases				
Previous history of mild malaria*			300	56,1
Clinical Malaria	−	−	113	21,1
Asymptomatic infection	−	−	122	22,8
Parasites in clinical malaria or				
asymptomatic infection				
*P. falciparum*	−	−	106	45.7
*P. vivax*	−	−	110	47.5
both	−	−	16	6.9

* these individuals were not infected at the moment of blood collection but recorded as having previously mild malaria; Controls were healthy individuals with no history of previous malaria; For some statistical analysis we have grouped clinical malaria patients, asymptomatic infected individuals and individuals with previous history of malaria (*any_malaria* group) and clinical malaria patients previous history of mild malaria (*clinical_malaria* group).

### Cases Definitions and Parasite Identification

Three definitions of malaria cases were used for the association analysis: clinical malaria (mild, n = 113), asymptomatic infected individuals (n = 122) and individuals with a previous history of mild malaria (non-asymptomatic infection), but not infected at the moment of blood collection (n = 300). Clinical malaria was defined in accordance to the guidelines of the World Health Organization (WHO) for the American region and the Brazilian National Malaria Control Programme (PNCM) including symptoms associated to malaria (i.e., fever, chills or diaphoresis) and a positive thick smear or rapid diagnostic test (RDT). Asymptomatic *Plasmodium*-infected cases were defined as an individual without symptoms for malaria within 30 days before or after the blood collection, but with a positive thick smear and/or PCR, following the recommendations of the Brazilian consensus group for studies of asymptomatic individuals.

For each sample collected, a thick smear was prepared and stained with Giemsa using the National Guidelines that was examined by a certified expert using 200 microscope fields under immersion oil. All positive samples were confirmed by another individual blinded to the previous results and the infecting *Plasmodium* species was identified by PCR according to published protocols [17]. There was an even balance between *P. falciparum* and *P.vivax* (Table 1).

### Sample Preparation and Genotyping

The sample collection consisted of 535 cases (Barcelos n = 494, Santa Isabel n = 41) and 113 healthy controls with no previous history of malaria (Barcelos n = 102, Santa Isabel n = 11). DNA samples were prepared by extraction from 300 µl of total blood using a commercial kit following the manufacturer’s protocol (Promega®). Genomic DNA samples underwent whole genome amplification by Primer Extension Pre-amplification (PEP) before genotyping on a Sequenom® MassArray genotyping platform (http://www.sequenom.com) [29], [30]. All samples underwent genotyping on the same instrument resulting in low rates of missing genotyping data. Sixty-four malaria candidate SNPs were genotyped, including: Haemoglobin variant S (HbS) (rs334), and an ABO blood group SNP that defines groups B and non-B (rs8176746). The full list can be found in Table S1.

### Statistical Methods

Association studies were performed comparing different groups: a) *any_malaria* group (clinical malaria patients, asymptomatic infected individuals, and individuals with a previous history of malaria) with the *never_malaria* group (control); b) *clinical_malaria* group (current or previous mild malaria) with the *never_malaria* group; c) *asymptomatic* infection group with the *never_malaria* group and d) *clinical_malaria* group (current or previous mild malaria) with *asymptomatic* infection group.

Genotypic deviations from the Hardy-Weinberg equilibrium (HWE) were assessed using a chi-square statistical test. SNPs were excluded from the analysis if there was at least 10% of the genotype calls missing or a significant deviation from the HWE (p<0.0001). A case-control association analysis using SNP alleles and genotypes was undertaken by logistic regression and included site (Barcelos or Santa Isabel do Rio Negro) and gender as covariates. In this approach, the SNP of interest was modeled assuming several related genotypic mechanisms (additive, dominant, recessive, heterozygous advantage and general models) and the minimum *P*-values from these correlated tests are reported. Haplotypes were estimated using an expectation-maximization algorithm [31], and score tests [32] were applied to assess the level of evidence for both global and individual haplotype associations with malaria. Linkage disequilibrium was estimated using the pairwise-SNP r^2^ and D-prime metrics.

Population structure or differences in allele frequencies between sub-populations can lead to false positive associations. Differences in allele frequencies were estimated between Barcelos and Santa Isabel do Rio Negro using an *Fst* metric [33], where values close to zero imply no difference, and values close to one imply complete differentiation between locations.

Statistical analysis of SNPs on the X chromosome was performed for each gender separately and, where applicable, the results were pooled using meta-analytic techniques. All analyses were performed using the R statistical package (http://www.r-project.org). Performing multiple statistical tests lead to an inflation in the occurrence of false positives and, by using a permutation approach that accounted for correlation between markers and tests, the estimated p-value cut-off of 0.003 was considered statistically significant.

## Results

**Figure 1:**
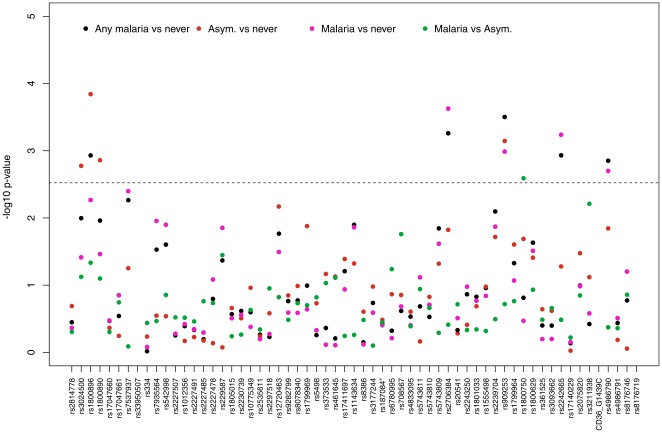
Minimum p-values from tests of association for the autosomal SNPs.* any malaria: *Any_malaria* group (clinical malaria, asymptomatic infection and previous history of malaria); asym: *Asymptomatic* group (individuals with asymptomatic infection); malaria: *Clinical_Malaria* group (current or previous history of mild malaria); never: *never_malaria* group (no history of previous malaria); genotypic tests of dominant, recessive, general, heterozygous advantage, and additive models, adjusted for gender; the dashed line represents a *p-value* of 0.003.

Overall, nine SNPs were excluded from the analysis because there was either a minor allele frequency of less than 1% (rs33950507, rs2227507, rs12720463, rs9282799, rs8386, rs5743809, hCD36_G1439C) or due to a high rate of missing genotype calls (rs7935564, rs20541). Figure 1 shows the minimum p-values from the genotypic tests applied to the autosomal SNPs.

There were five significant results for those with *any_malaria* versus *never_malaria* group: rs1800896– IL10-1082 (OR: 0.528, CI: 0.360–0.774; P = 0.0014), rs2706384 - IRF1 (OR: 1.881, 95% CI: 1.298–2.724; P = 0.0005), rs2242665 - CTL4 (OR: 0.595, 95% CI: 0.434–0.816; P = 0.0012), rs4986790 - TLR4 (OR: 0.274, 95% CI: 0.124–0.604; P = 0.0014) and rs909253 - LTA+252 (OR: 0.343, 95% CI: 0.182–0.647; P = 0.0009) (see Table 2). Four of these SNPs were also significant when analyzing *clinical_malaria* versus *never_malaria* group: rs2706384 - IRF1 (OR: 2.023, 95% CI: 1.371–2.987; P = 0.0002), rs2242665 - CTL4 (OR: 0.564, 95% CI: 0.406–0.784; P = 0.0006), rs4986790 - TLR4 (OR: 0.271, 95% CI: 0.116–0.633; P = 0.002) and rs909253 - LTA+252 (OR: 0.366, 95% CI: 0.192–0.699; P = 0.001). When analyzing *clinical_malaria* versus *asymptomatic* infection group, associations were observed with the TNF-376 promoter SNP (rs1800750, OR:0.086, 95% CI: 0.016–0.473; P = 0.0026), previously associated in a number of other malaria studies. (see [11] for a review). The LTA and IL10 SNPs were also significant when analyzing the *asymptomatic* infection versus *never_malaria* groups: rs909253 - LTA+252 (OR: 3.508, 95% CI: 1.641–7.502; P = 0.0007), rs1800896 - IL10-1082 (OR: 0.280, 95% CI: 0.142–0.552; P = 0.0001), rs3024500 (OR: 0.418, 95% CI:0.236–0.739; P = 0.0017) and rs1800890 - IL10-3533 (OR: 0.280, 95% CI: 0.194–0.713; P = 0.0001). There was a high linkage disequilbrium (LD) between the three IL10 polymorphisms (minimum pairwise D’  = 0.85). A haplotype analysis of these three polymorphisms (rs3024500, rs1800896, rs1800890) revealed that those with the GCT allelic combination (∼10% frequency in population) were at a lower risk of any form of malaria (OR: 0.40–0.63, 95% CI: 0.2–0.9) when compared to the common ATA combination (>83% frequency in population; see Table 3).

**Table 2:** Allele frequencies and tests of association for the most significant SNPs.

Comparison		SNP	Ancestral/	Derived	Minor	MAF	MAF	Contrast	OR	LCL	UCL	*p-value*
groups			Ref. allele	allele	allele	Controls	Cases					
*Asymptomatic*	*Never_malaria*	rs909253 - LTA+252	A	G	A	0.476	0.519	Recessive	3.508	1.641	7.502	0.0007
		rs1800896 -IL10-1082	T	C	T	0.212	0.094	Dominant	0.280	0.142	0.552	0.0001
		rs1800890 -IL-10-3533	A	T	A	0.167	0.075	Additive	0.372	0.194	0.713	0.0014
		rs3024500–IL10	G	A	A	0.207	0.107	Additive	0.418	0.236	0.739	0.0017
*Any_malaria*	*Never_malaria*	rs909253– LTA+252	A	G	G	0.524	0.468	Dominant	0.343	0.182	0.647	0.0009
		rs1800896 -IL10-1082	T	C	T	0.212	0.127	Additive	0.528	0.360	0.774	0.0014
		rs2706384 -IRF1	G	T	T	0.273	0.397	Additive	1.881	1.298	2.724	0.0005
		rs2242665 - CTL4	C	T	C	0.449	0.383	Additive	0.595	0.434	0.816	0.0012
		rs4986790 - TLR4	A	G	A	0.058	0.015	Additive	0.274	0.124	0.604	0.0014
*Clinical_Malaria*	*Never_malaria*	rs909253– LTA+252	A	G	G	0.524	0.465	Dominant	0.366	0.192	0.699	0.0010
		rs2706384 - IRF1	G	T	T	0.273	0.397	Additive	2.023	1.371	2.987	0.0002
		rs2242665 - CTL4	C	T	T	0.449	0.372	Additive	0.564	0.406	0.784	0.0006
		rs4986790 -TLR4	A	G	A	0.058	0.015	Additive	0.271	0.116	0.633	0.0020
*Clinical_Malaria*	*Asymptomatic*	rs1800750 -TNF-376	G	A	G	0.080	0.022	Recessive	0.086	0.016	0.473	0.0026

*Any_*malaria group consisted of: clinical malaria, asymptomatic infection and previous history of malaria; *Asymptomatic* group: asymptomatic infection; *Clinical_Malaria* group: clinical mild malaria (current or previous mild malaria); *Never_malaria* group: no history of malaria; Ref: reference; MinA: minor allele; MajA: major allele; MAF: minor allele frequency; HWEP: Hardy-Weinberg p-value; OR: odds ratio; 95% Confidence interval (LCL- UCL).

Analysis of the four polymorphisms on the X-chromosome revealed no significant associations (Table S2). These included the G6PD+202A allele, also referred to as A^−^, which is a deficiency surrogate that occurs at a low frequency in our population (<3%). Two other candidate polymorphisms with a strong presence in the malaria literature, type HbS and B blood groups, were present at low frequencies in our population (<4%) and, therefore, lacked the power to detect an association (P>0.03; see Table 4) in this study. It was interesting to note that in our population the null allele of the Duffy antigen was present at a frequency of ∼10% (Table 4).

**Table 3:** Haplotype analysis of IL10 gene.

Comparison		Haplotype	Hap-score	*p-value*	Controls	Cases	OR	LCL	UCL
groups					Freq.	Freq.			
*Any_malaria*	*Never_malaria*	GCT	−2.697	0.007	0.162	0.097	0.573	0.385	0.852
		GCA	−1.582	0.114	0.041	0.022	0.457	0.202	1.032
		GTA	1.299	0.194	0.005	0.017	3.337	0.449	24.80
		ATA	2.363	0.018	0.784	0.849	1.000	−	−
		global-stat = 12.459, df = 4, P = 0.0142							
*Asymptomatic*	*Never_malaria*	GCT	−2.894	0.004	0.162	0.069	0.406	0.221	0.744
		GCA	−1.468	0.142	0.041	0.017	0.342	0.101	1.158
		ATA	2.846	0.004	0.784	0.887	1.000	−	−
		global-stat = 12.127, df = 3, P = 0.0070							
*Clinical_Malaria*	*Never_malaria*	GCT	−2.225	0.026	0.162	0.105	0.625	0.416	0.938
		GCA	−1.380	0.168	0.041	0.024	0.492	0.212	1.138
		GTA	1.428	0.153	0.005	0.018	3.887	0.502	30.11
		ATA	1.903	0.057	0.784	0.839	1.000	−	−
		global-stat = 9.7201, df = 4, P = 0.0454							
*Clinical_Malaria*	*Asymptomatic*	GCT	1.599	0.110	0.069	0.105	1.579	0.914	2.728
		GCA	0.578	0.562	0.017	0.024	1.465	0.481	4.457
		GTA	0.394	0.694	0.014	0.018	1.306	0.391	4.356
		ATA	−1.728	0.084	0.887	0.839	1.000	−	−
		global-stat = 3.2488, df = 4, P = 0.5171							

*Any_*malaria group consisted of: clinical malaria, asymptomatic infection and previous history of malaria; *Asymptomatic* group: asymptomatic infection; *Clinical_Malaria* group: clinical mild malaria (current or previous mild malaria); *Never_malaria* group: no history of malaria: Haplotypes are for rs3024500, rs1800896,and rs1800890; Freq: frequency; OR: odds ratio; 95% Confidence interval (LCL- UCL); df : degrees of freedom.

There was no evidence of population structure effects from the two locations on the association analysis. First, the *Fst* values across all markers were close to zero (median 0.0009, range 0 to 0.0063; see Supplementary Table S3 for values). Second, by removing the data of Santa Isabel do Rio Negro from the analysis, the association hits were identical, but with a lower precision on the odds ratio estimates. Supplementary Tables S4, S5, S6, S7 display data on allele frequencies and tests of association between groups studied in this work.

**Table 4:** Odds ratios for the Duffy antigen, HbS (rs334), ABO, and G6PD-202.

Comparison		SNP	Ancestral/	Derived	Minor	MAF	MAF	Contrast	OR	LCL	UCL	*p-valu*e
groups			ref. allele	allele	allele	Controls	Cases					
*Any_malaria*	*Never_malaria*	rs2814778 - DARC	T	C	C	0.106	0.098	CC vs TT/TC	0.434	0.082	2.276	0.357
		rs334–HbS (HBB)	T	A	A	0.014	0.017	TA vs TT	1.033	0.286	3.723	0.961
		rs8176746 - ABO	G	T	G	0.037	0.027	TG vs other	0.532	0.225	1.260	0.169
		rs1050828–G6PD+202	C	T	T	0.005	0.021	Additive T	5.235	0.758	36.179	0.093
*Asymptomatic*	*Never_malaria*	rs2814778 - DARC	T	C	C	0.106	0.087	CC vs TT/TC	0.216	0.018	2.525	0.204
		rs334–HbS (HBB)	T	A	A	0.014	0.023	TA vs TT	1.526	0.334	6.964	0.582
		rs8176746 - ABO	G	T	G	0.037	0.040	TG vs other	0.923	0.328	2.600	0.880
		rs1050828–G6PD+202	C	T	T	0.005	0.043	Additive T	8.663	1.122	66.908	0.038
*Clinical_Malaria*	*Never_malaria*	rs2814778 - DARC	T	C	C	0.106	0.101	CC vs TT/TC	0.491	0.090	2.667	0.434
		rs334–HbS (HBB)	T	A	A	0.014	0.015	TA vs TT	0.864	0.226	3.300	0.832
		rs8176746 - ABO	G	T	G	0.037	0.024	TG vs other	0.398	0.157	1.010	0.063
		rs1050828–G6PD+202	C	T	T	0.005	0.015	Additive T	4.177	0.586	29.754	0.154
*Clinical_Malaria*	*Asymptomatic*	rs2814778 - DARC	T	C	C	0.087	0.101	CC vs TT/TC	1.194	0.674	2.114	0.539
		rs334–HbS (HBB)	T	A	A	0.023	0.015	TA vs TT	0.595	0.201	1.763	0.365
		rs8176746 - ABO	G	T	G	0.040	0.024	TG vs other	0.515	0.220	1.206	0.139
		rs1050828–G6PD+202	C	T	T	0.043	0.015	Additive T	0.359	0.143	0.905	0.030

*Any_malaria* group consisted of: clinical malaria, asymptomatic infection and previous history of malaria; *Asymptomatic* group: asymptomatic infection; *Clinical_Malaria* group: clinical mild malaria (current or previous history of mild malaria ); *Never_malaria* group: no history of malaria; Ref: reference; MAF: minor allele frequency; OR  =  odds ratio; 95% Confidence interval (LCL, UCL).

## Discussion

This genetic association study was designed to correlate the presence of various host gene polymorphisms within a Brazilian Amazonian population with the clinical presentation of malaria for the purpose of identifying candidate genes whose functions could impact disease progression. The results showed, for the first time, an association between alleles of CTL4 gene with malaria. Within the MHC class III region, the SNP (rs2242665) located in the CTL4 gene, displayed a significant association with reduced risk for clinical (mild) malaria. This gene encodes for a possible sodium-dependent transmembrane transport protein involved in the uptake of choline by cholinergic neurons. As the MHC class III region has a complex haplotype structure with long-range LD patterns, this finding could arise from a functional variant in high linkage with this SNP.

Polymorphic variability in the innate immune response gene IL-10 also showed a strong haplotype risk association (OR <0.7 for the GCT haplotype, see Table 3) on both asymptomatic infection and clinical (mild) malaria. When analysed at the level of individual SNPs, an association was discovered for those individuals displaying IL10 - 1082 with a reduced risk for malaria symptoms. Previous studies in a Kenyan population reported similar relationships between common African IL10 promoter variants (−1082A/G (this study), −819T/C, and −592A/C), and protection against severe malarial anaemia and an increased production of IL10 [34]. The absence of immunoassay data within this study and severe malaria phenotypes observed in the participants was a limitation to demonstrating the role of IL10 in mitigating *Plasmodium* infections.

Two other associations with cytokines were identified, both within MHC class III region, that included TNF and the lymphotoxin alpha (LT-α/LTA) and beta (LT-ß/LTB) genes, which are closely related. The TNF and LTA genes are implicated in the host defense and pathogeneses of severe malaria [5], [35]. An intronic SNP in LTA (rs909253) was associated with protection from clinical (mild) malaria. Previous studies showed no significant risk associated with LTA in a Sri Lankan population [36] or cohorts in Kenya and Malawi [11]. These disparities of association could be due to differences in the genetic background between the Brazilian population with those of the Sri Lankan and African populations.

In addition, the TNF-376 promoter SNP (rs1800750) was identified to have an association with clinical malaria (Table 2). An association was previously found for this SNP with an increased risk for cerebral malaria in Kenya and Gambia, conferring an allele-specific binding of the transcription factor OCT-1([37], [38]; see [11] for a review). This observation adds support to the current dogma that the TNF locus is important for malaria pathogenesis. However, it is necessary to corroborate these findings with a large scale epidemiological and immunological functional study.

Other cytokine-related findings include one SNP (rs2706384) in the Interferon Regulatory Factor 1 gene (IRF1), which was associated with an increased risk for clinical malaria. IRF1 is a critical mediator of IFN-γ activity and is crucial in both innate and adaptative immune responses against *P. falciparum* [39]. Our finding are consistent with a previously reported association between polymorphisms in the IRF1 and the control of *P. falciparum* infection both in healthy adults and children displaying severe and uncomplicated malaria [40]. Moreover, this gene is located in the 5q31 region, which has been shown to be associated with *P. falciparum* infection levels [32], [33].

Finally, a polymorphism was seen in the TLR4 gene (rs4986790) that was associated with reduced risk for clinical malaria. Previous studies in an Amazonian population reported no association between TLR4 and mild malaria [20], [22]. However, the absence of an apparent association may be due to the lower minor allele frequencies and smaller samples sizes of those studies. Unlike the results reported here, two studies in Ghana have revealed frequent polymorphisms at TLR4 that conferred an increased risk of severe malaria [12] and clinical manifestations of malaria during pregnancy [13]. The absence of severe malaria in Brazil could explain the differences in association results. Together, the genetic data support a role for TLR4 in modulating the presentation of malaria symptoms.

Overall, this study represents the first association study from an Amazonian population involving a large number of host genetic polymorphisms with susceptibility or resistance to *Plasmodium* infection and malaria outcomes. To understand which are the real causal variants, re-sequencing of LTA, TNF and CTL4 genes and the surrounding MHC class III in a range of populations will be necessary to assist the design of large scale epidemiological studies. Previous candidate polymorphisms have arisen mostly from studies in an African setting, where the linkage disequilibrium or correlation structure between SNPs, the absence of *Plasmodium vivax*, endemicity, and disease severity complicate their relevancy to Amazonian populations. For example, the data analysis from our study revealed no significant associations for gene polymorphisms of the sickle-cell trait, blood-related polymorphisms and G6PD A^-^ surrogate, which display inherited innate resistance to malaria. These results were not unexpected, given that the low frequencies of these alleles in the Brazilian population together with the current sample size that restricted the resolving power of the analysis to detect associations.

The issue of malaria disease association mapping and its implications for disease management in multiple geographic locations remains a major challenge confronting the field. The differences highlighted here and the association of specific SNP polymorphisms with clinical malaria in the Amazon basin support the need for additional studies. The results suggest a need for associations studies with more dense mapping of candidate genes, especially CTL4 and IL-10, along with other genes from the major histocompatibility complex region to identify additional functional variants most relevant for malaria in Amazonian populations.

## Supporting Information

Table S1 **List of all polymorphisms genotyped in this study.**
(XLS)

Table S2 **X chromosome analysis.**
(XLS)

Table S3 **Allele frequencies differences between Santa Isabel do Rio Negro and Barcelos.**
(XLS)

Table S4 **Allele frequencies and test of association -** ***Any_malaria*** **versus** ***never_malaria*** **group.**
(XLS)

Table S5 **Allele frequencies and test of association -** ***Asymptomatic*** **versus** ***never_malaria*** **group.**
(XLS)

Table S6 **Allele frequencies and test of association -** ***Clinical_malaria*** **versus** ***never_malaria*** **group.**
(XLS)

Table S7 **Allele frequencies and test of association -** ***Clinical_malaria*** **versus** ***asymptomatic*** **group.**
(XLS)
